# Supplementary material for: Zika virus modulates human fibroblasts to enhance transmission success in a controlled lab-setting
Source: Commun Biol. 2025 Jan 30;8:139. doi: 10.1038/s42003-025-07543-9 (PMC11782651; doi:10.1038/s42003-025-07543-9)
Supplement: Supplementary file 1 — Supplementary Information [file 42003_2025_7543_MOESM1_ESM.pdf]

Supplementary Information for

Zika virus modulates human fibroblasts to enhance transmission success in a controlled lab-setting

This file includes:

Supplementary Figs 1 to 7

Supplementary Tables 1 to 5

a

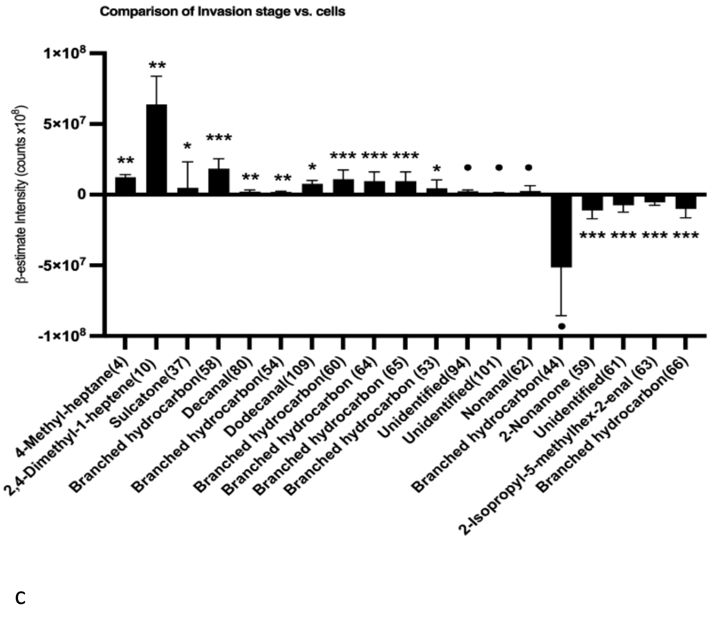
Comparison of Invasion stage vs. cells
β-estimate Intensity (counts x10^8)
1×10^8
5×10^7
0
-5×10^7
-1×10^8
4-Methyl-heptane(4)
2,4-Dimethyl-1-heptene(10)
Sulcatone(37)
Branched hydrocarbon(58)
Decanal(80)
Branched hydrocarbon(54)
Dodecanal(109)
Branched hydrocarbon(60)
Branched hydrocarbon (64)
Branched hydrocarbon (65)
Branched hydrocarbon (53)
Unidentified(94)
Unidentified(101)
Nonanal(62)
Branched hydrocarbon(44)
2-Nonanone (59)
Unidentified(61)
2-Isopropyl-5-methylhex-2-enal (63)
Branched hydrocarbon(66)

b

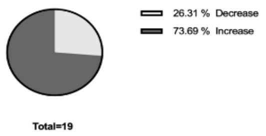
26.31 % Decrease
73.69 % Increase
Total=19

c

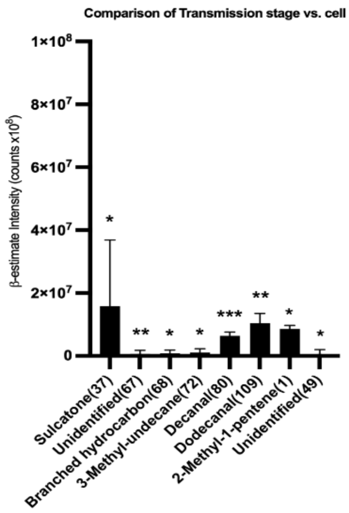
Comparison of Transmission stage vs. cell
β-estimate Intensity (counts x10^8)
1×10^8
8×10^7
6×10^7
4×10^7
2×10^7
0
Sulcatone(37)
Unidentified(67)
Branched hydrocarbon(68)
3-Methyl-undecane(72)
Decanal(80)
Dodecanal(109)
2-Methyl-1-pentene(1)
Unidentified(49)

d

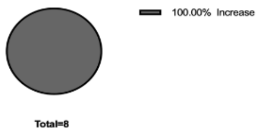
100.00% Increase
Total=8

e

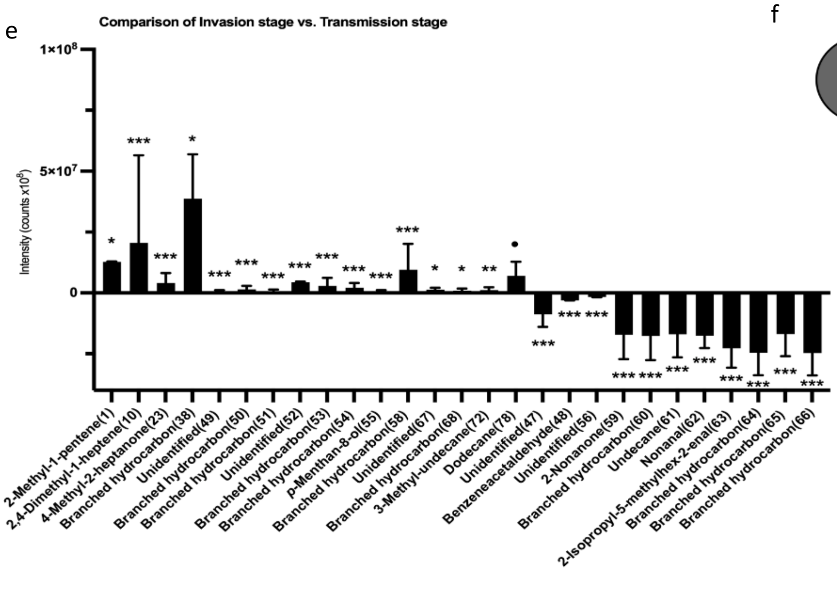
Comparison of Invasion stage vs. Transmission stage
Intensity (counts x10^8)
1×10^8
5×10^7
0
2-Methyl-1-pentene(1)
2,4-Dimethyl-1-heptene(10)
4-Methyl-2-heptanone(23)
Branched hydrocarbon(38)
Unidentified(49)
Branched hydrocarbon(50)
Branched hydrocarbon(51)
Unidentified(52)
Branched hydrocarbon(53)
Branched hydrocarbon(54)
p-Menthan-8-ol(55)
Branched hydrocarbon(58)
Unidentified(67)
Branched hydrocarbon(68)
3-Methyl-undecane(72)
Dodecane(78)
Unidentified(47)
Benzeneacetaldehyde(48)
Unidentified(56)
2-Nonanone(59)
Branched hydrocarbon(60)
Undecane(61)
Nonanal(62)
2-Isopropyl-5-methylhex-2-enal(63)
Branched hydrocarbon(64)
Branched hydrocarbon(65)
Branched hydrocarbon(66)

f

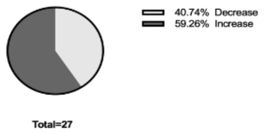
40.74% Decrease
59.26% Increase
Total=27

g

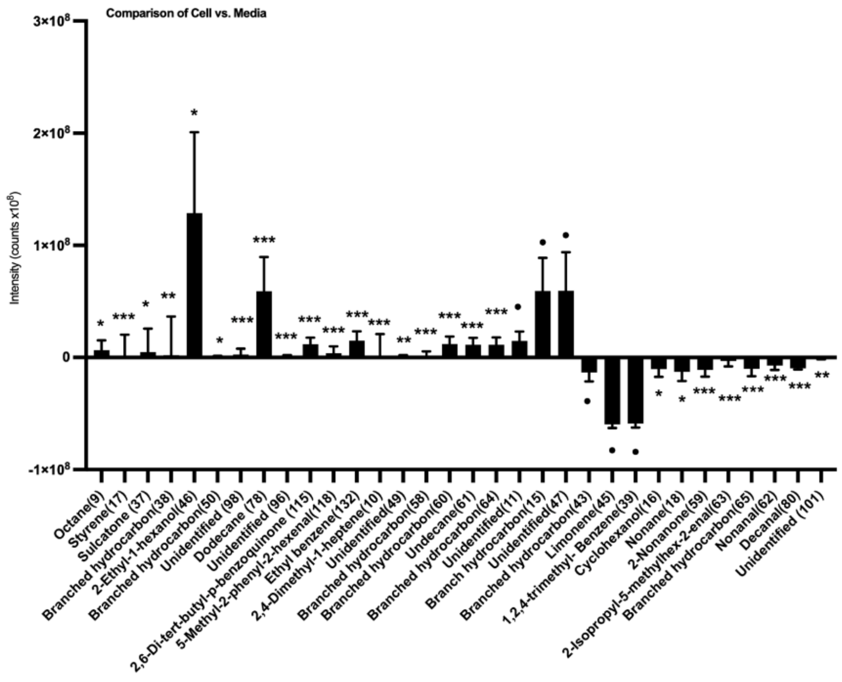
Comparison of Cell vs. Media
Intensity (counts x10^8)
3×10^8
2×10^8
1×10^8
0
-1×10^8
Octane(9)
Styrene(17)
Sulcatone (37)
Branched hydrocarbon(38)
2-Ethyl-1-hexanol(46)
Branched hydrocarbon(50)
Unidentified (98)
Dodecane (78)
Unidentified (96)
2,6-Di-tert-butyl-p-benzoquinone (115)
5-Methyl-2-phenyl-2-hexenal(118)
Ethyl benzene(132)
2,4-Dimethyl-1-heptene(10)
Unidentified(49)
Branched hydrocarbon(58)
Branched hydrocarbon(60)
Undecane(61)
Branched hydrocarbon(64)
Unidentified(11)
Branch hydrocarbon(15)
Unidentified(47)
Branched hydrocarbon(43)
Limonene(45)
1,2,4-trimethyl- Benzene(39)
Cyclohexanol(16)
Nonane(18)
2-Nonanone(59)
2-Isopropyl-5-methylhex-2-enal(63)
Branched hydrocarbon(65)
Nonanal(62)
Decanal(80)
Unidentified (101)

h

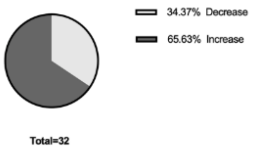
34.37% Decrease
65.63% Increase
Total=32

i

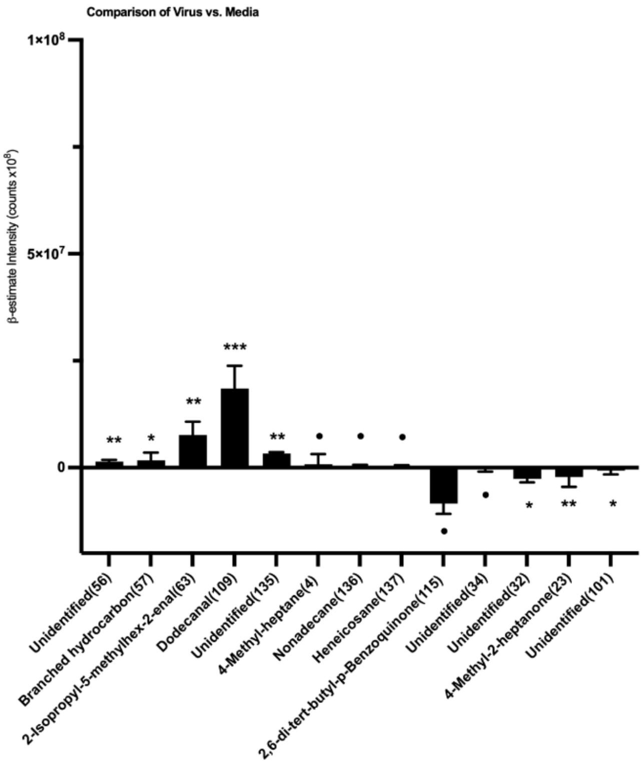
Comparison of Virus vs. Media
β-estimate Intensity (counts x10^8)
1×10^8
5×10^7
0
Unidentified(56)
Branched hydrocarbon(57)
2-Isopropyl-5-methylhex-2-enal(63)
Dodecanal(109)
Unidentified(135)
4-Methyl-heptane(4)
Nonadecane(136)
Heneicosane(137)
2,6-di-tert-butyl-p-Benzoquinone(115)
Unidentified(34)
Unidentified(32)
4-Methyl-2-heptanone(23)
Unidentified(101)

j

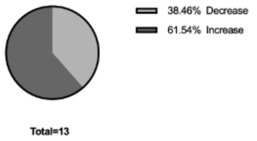
38.46% Decrease
61.54% Increase
Total=13

Supplementary Fig. 1 | Volatile organic compounds (VOCs) whose amounts differ significantly in pairwise comparisons.

Fibroblast culture cells infected with ZIKV at invasion or transmission stage versus uninfected fibroblast culture cells (a-d); fibroblast culture cells infected with ZIKV at invasion versus transmission (e-f); uninfected fibroblast culture cells versus media (g-h); virus in culture media versus media (i-j). Significant effects were determined using GLMM models (lmer) [in all experiments: n=30/ Experimental replication]; Bars represented by *β*-estimation generated by the mixed model ± SE (*β*-lmer ± SE); asterisks denote significant differences (*$p < 0{\cdot}05$; **$p < 0{\cdot}01$; ***$p < 0{\cdot}001$). All statistical model descriptions are provided in Supplementary Table 1, and the raw data can be found in Supplementary Data file 1.

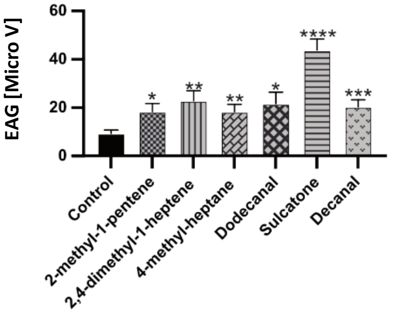

Supplementary Fig. 2 | Electroantennographic (EAG) responses of the *Ae. aegypti* female mosquitoes to six commercially available synthetic VOCs. EAG measurement of the *Aedes* in response to the ZIKV blends single components were measured and analysed. Significant effects were determined using GLMM models (lmer) [in all experiments: n=30 per experimental replication]; Bars represented by β-estimation generated by the mixed model ± SE (β-lmer ± SE); asterisks denote significant differences (*$p < 0{\cdot}05$; **$p < 0{\cdot}01$; ***$p < 0{\cdot}001$). All statistical model descriptions are provided in Supplementary Table 2, and the raw data can be found in Supplementary Data file 1.

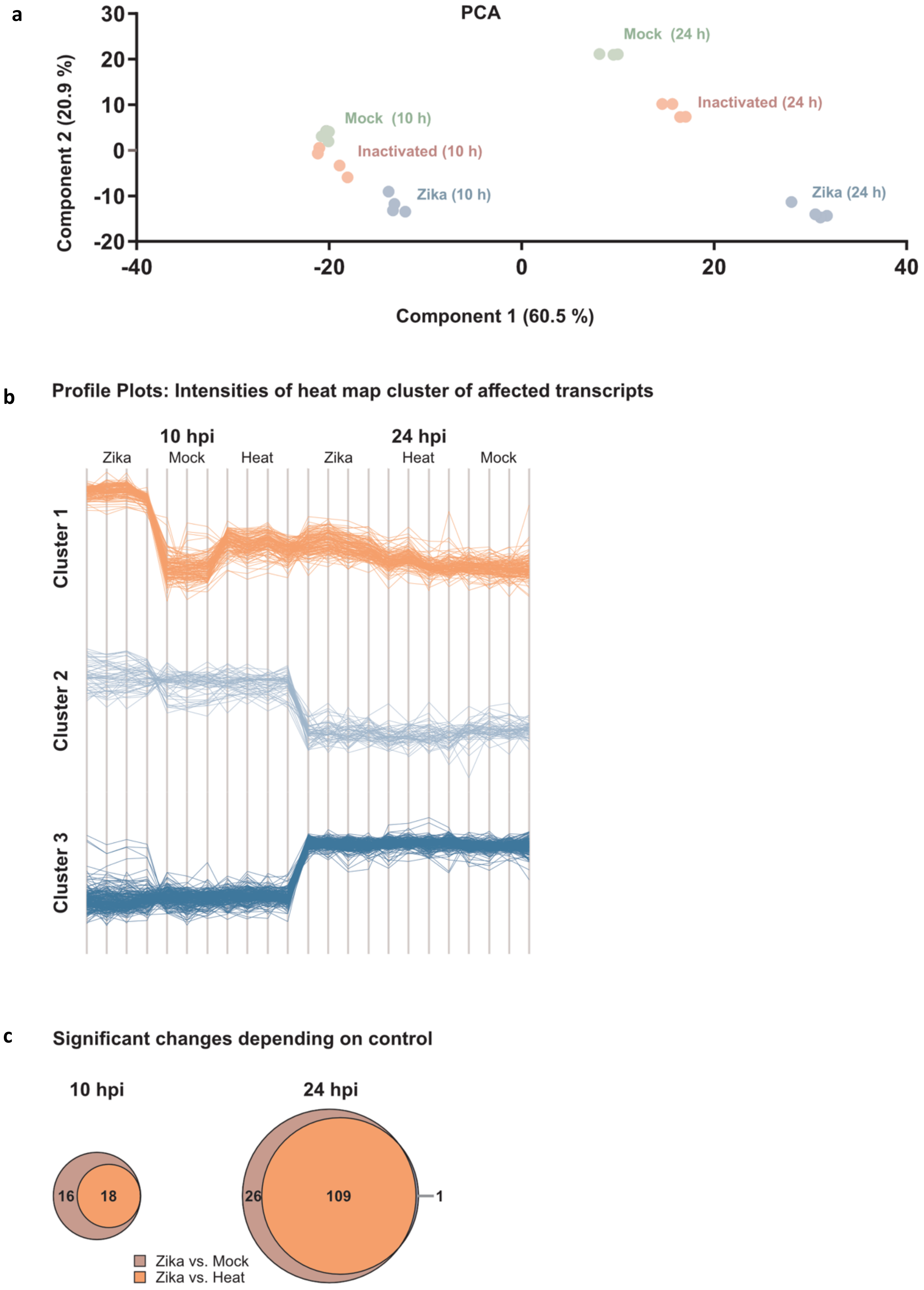
a
PCA
Component 2 (20.9 %)
Component 1 (60.5 %)
30
20
10
0
-10
-20
-40
-20
0
20
40
Mock (24 h)
Inactivated (24 h)
Mock (10 h)
Inactivated (10 h)
Zika (10 h)
Zika (24 h)
b
Profile Plots: Intensities of heat map cluster of affected transcripts
10 hpi
24 hpi
Zika
Mock
Heat
Zika
Heat
Mock
Cluster 1
Cluster 2
Cluster 3
c
Significant changes depending on control
10 hpi
24 hpi
16
18
26
109
1
Zika vs. Mock
Zika vs. Heat

d

Transcripts affected by Zika virus infection (10 hpi or 24 hpi)

Significant changes compared to Mock and Heat-Inavtivated control

No	Gene Name	10 hpi		24 hpi	
		Mock	Heat	Mock	Heat
1	AC048341.2	0.50	-0.56	-1.01	-1.42
2	AGRN	0.10	0.08	1.57	1.08
3	AL445490.1	1.83	1.29	3.05	1.64
4	ANGPTL1	0.14	0.23	2.34	1.77
5	APOBEC3F	0.19	0.33	1.12	1.16
6	APOBEC3G	0.51	0.69	2.08	1.82
7	APOL1	0.32	0.40	2.42	1.90
8	APOL2	0.71	0.55	1.66	1.34
9	APOL3	0.59	0.71	1.71	1.54
10	APOL6	0.48	0.47	1.77	1.33
11	ATF3	0.05	0.20	1.70	1.58
12	BATF2	2.10	1.90	4.11	3.08
13	BST2	0.79	0.66	4.33	2.40
14	CASP1	-0.04	0.27	1.28	1.15
15	CMPK2	1.79	1.32	4.83	3.55
16	DDO	0.13	0.27	1.78	1.45
17	DDX58	1.88	1.27	3.54	2.03
18	DDX60	0.68	0.52	2.76	1.50
19	DDX60L	0.72	0.56	2.49	1.68
20	DHX58	0.82	0.57	2.57	1.59
21	DTX3L	1.09	0.75	2.46	1.41
22	EIF2AK2	0.71	0.55	2.02	1.21
23	EPSTI1	1.41	0.86	3.53	1.89
24	GBP1	0.84	0.68	2.07	1.74
25	GBP5	1.01	0.86	2.43	2.13
26	GCA	0.40	0.70	1.89	1.53
27	GCH1	0.29	0.22	1.52	1.05
28	GMPR	0.29	0.59	2.61	2.02
29	HELZ2	1.85	1.20	3.24	1.82
30	HERC5	0.84	0.76	3.41	2.69
31	HERC6	1.45	1.00	4.88	2.36
32	HES4	0.14	0.01	1.68	1.36
33	IFI27	0.93	0.67	4.37	2.16
34	IFI35	0.63	0.40	3.12	2.02
35	IFI44	1.16	0.83	3.23	1.66
36	IFI44L	1.62	1.01	4.83	2.23
37	IFI6	1.25	0.81	4.42	1.96
38	IFIH1	1.94	1.35	3.94	2.15
39	IFIT1	2.32	1.44	4.05	2.05
40	IFIT2	2.88	2.13	4.19	3.33
41	IFIT3	1.96	1.40	3.92	2.29
42	IFIT5	0.85	0.72	1.63	1.18
43	IFITM1	1.04	0.83	4.09	2.16
44	IFITM3	0.12	0.11	1.49	1.02
45	IL15RA	0.21	0.27	1.65	1.44
46	IRF7	1.22	1.00	3.08	1.64
47	ISG15	1.93	1.19	4.83	2.15
48	ISG20	0.66	0.38	2.61	2.28
49	LAP3	0.28	0.20	1.72	1.23
50	LGALS9	0.50	0.59	3.14	2.15
51	MX1	1.51	0.89	4.22	2.59
52	MX2	2.11	1.27	4.99	2.58
53	MYD88	0.64	0.57	1.84	1.41
54	NEDD9	-0.04	-0.08	1.06	1.26
55	NLRC5	0.52	0.51	1.52	1.20
56	NMI	0.40	0.42	1.78	1.45
57	OAS1	2.18	1.21	4.76	2.22
58	OAS2	1.98	1.20	4.43	2.06
59	OAS3	1.30	0.91	4.30	2.26
60	OASL	2.64	1.65	4.63	3.50
61	ODF3B	0.39	0.56	1.94	1.26
62	PARP10	0.66	0.51	2.28	1.35
63	PARP12	1.11	0.68	2.86	1.72
64	PARP14	0.73	0.56	3.16	1.95
65	PARP9	1.21	0.79	2.83	1.43
66	PLAAT4	0.18	0.29	1.56	1.34
67	PLEKHA4	0.48	0.52	2.19	1.72
68	PLSCR1	0.74	0.57	2.98	1.72
69	PMAIP1	0.20	0.14	1.32	1.08
70	PML	0.56	0.44	1.31	1.00
71	PNPT1	0.45	0.42	1.76	1.45
72	PPM1K	0.26	0.08	1.39	1.21
73	PSMB8	0.15	0.15	1.34	1.04
74	PSMB9	0.29	0.32	2.31	1.77
75	RNF213	0.22	0.20	1.36	1.15
76	RSAD2	2.53	1.48	5.94	4.29
77	RTP4	1.33	1.29	3.53	2.58
78	SAMD9	0.82	0.72	2.08	1.41
79	SAMD9L	0.72	0.64	2.27	1.50
80	SAMHD1	0.58	0.49	3.01	1.94
81	SECTM1	0.16	0.61	1.97	1.70
82	SHFL	0.84	0.69	2.82	1.81
83	SLC15A3	0.70	0.59	2.93	1.97
84	SLFN5	0.56	0.53	1.52	1.03
85	SP100	0.36	0.35	1.44	1.00
86	SP110	0.83	0.70	2.60	1.65
87	STAT1	0.55	0.39	2.12	1.32
88	STAT2	0.53	0.34	1.69	1.12
89	TAP1	0.62	0.46	2.31	1.67
90	TAP2	0.33	0.27	1.86	1.31
91	TDRD7	0.35	0.36	1.34	1.04
92	THEMIS2	1.55	1.27	2.97	2.31
93	TLR3	0.75	0.84	3.23	2.61
94	TMEM140	0.50	0.50	1.42	1.03
95	TNFSF10	1.41	0.93	3.41	3.06
96	TNFSF13B	0.66	0.83	2.84	2.26
97	TRANK1	0.43	0.41	2.00	1.59
98	TRIM14	0.68	0.41	2.63	1.58
99	TRIM21	0.96	0.78	2.07	1.57
100	TRIM22	0.38	0.37	1.99	1.46
101	TRIM25	0.63	0.51	1.80	1.18
102	TRIM5	0.32	0.28	1.30	1.07
103	TRIM69	0.68	0.49	1.99	1.39
104	UBA7	0.32	0.41	1.70	1.32
105	UBE2L6	0.41	0.42	2.46	1.79
106	UNC93B1	0.00	0.10	1.32	1.04
107	USP18	1.29	0.80	3.63	2.17
108	XAF1	1.07	0.62	2.53	1.30
109	ZNFX1	0.68	0.52	1.40	1.02

Supplementary Fig. 3 | Distinct effects of ZIKV on temporal gene regulation in human skin fibroblasts. (a) Principal component analysis (all transcripts; all experimental conditions). (b) Profile plots derived from heat map showing three major clusters of transcripts (main Fig. b). (c) Venn diagram depicting the overlap of ZIKV specific hits depending on heat inactivated ZIKV or Mock controls. (d) Table of transcripts with a significantly altered expression upon infection with

ZIKV, independent from control. The red colour indicates fold change. Transcripts highlighted in green were already affected at 10 hpi.

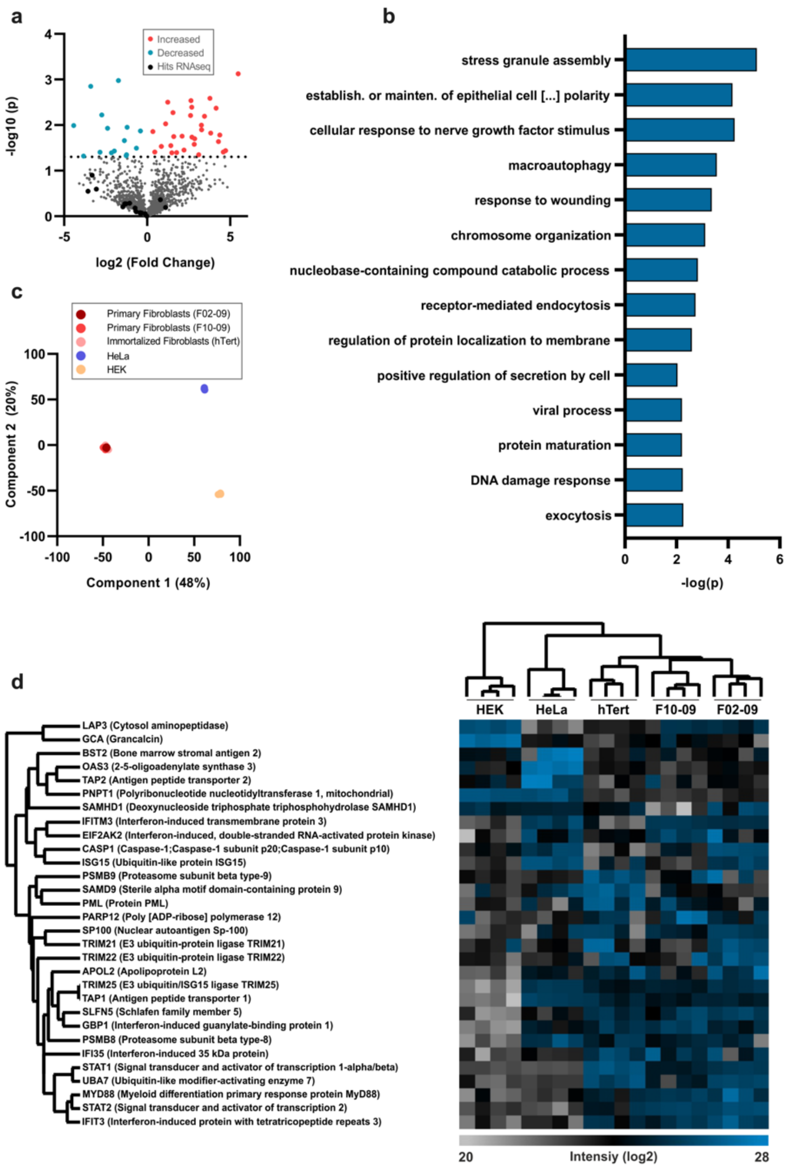

Supplementary Fig. 4 | Proteome analysis of ZIKV-infected and primary fibroblasts. LC-MS analysis was applied to compare ZIKV-infected to Mock-infected immortalised human dermal fibroblasts **(a,b)** as well as immortalised human dermal fibroblasts to primary fibroblasts **(c,d)**. **(a)** Volcano plots showing protein abundances in ZIKV-infected compared to those in Mock-infected samples (24 hpi, Student's t-tests, n=4 and n=2, respectively). Red/blue dots: proteins with significantly increased/decreased abundance upon ZIKV infection. Black dots: proteins with significantly altered expression on RNA level upon ZIKV infection according to RNAseq analysis. **(b)** Results of enrichment analysis (*Metascape*) showing processes (Gene Ontology of Biological Processes, $p \leq 0.05$) enriched among proteins affected by ZIKV infection. **(c)** Principal component

analysis (n=4) comparing the proteomics profiles of immortalized human dermal fibroblasts (hTert), primary human dermal fibroblasts (F02-09, F10-09) and two protein digest standards used as references (HeLa, HEK). **(d)** Hierarchical clustering (n=4) of protein abundance in immortalized fibroblasts, primary fibroblasts and references of proteins with significantly altered expression on RNA level upon ZIKV infection according to RNAseq analysis.

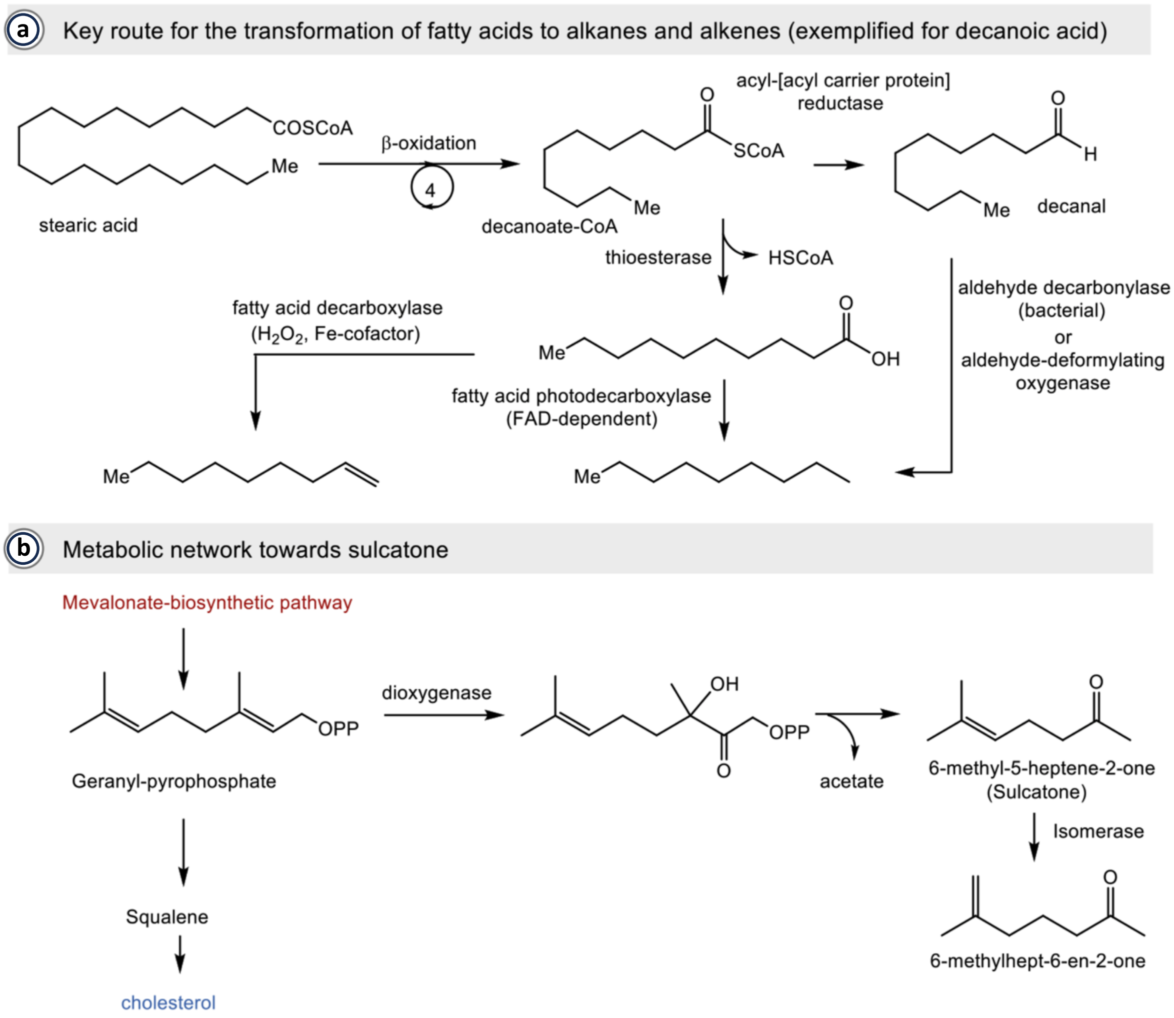

Supplementary Fig. 5 | Fatty acid metabolism that yields alkanes and alkenes by combinations of reductive steps and removal of the carboxylate terminus are summarized and exemplified for decanoate-SCoA. (a) The key transformation that supposedly is involved in the formation of volatiles reported. (b) Proposed biosynthesis of 6-methyl-5-heptene-2-one (Sulcatone) and 6-methylhept-6-en-2-one from GPP (mevalonate pathway of terpenes).

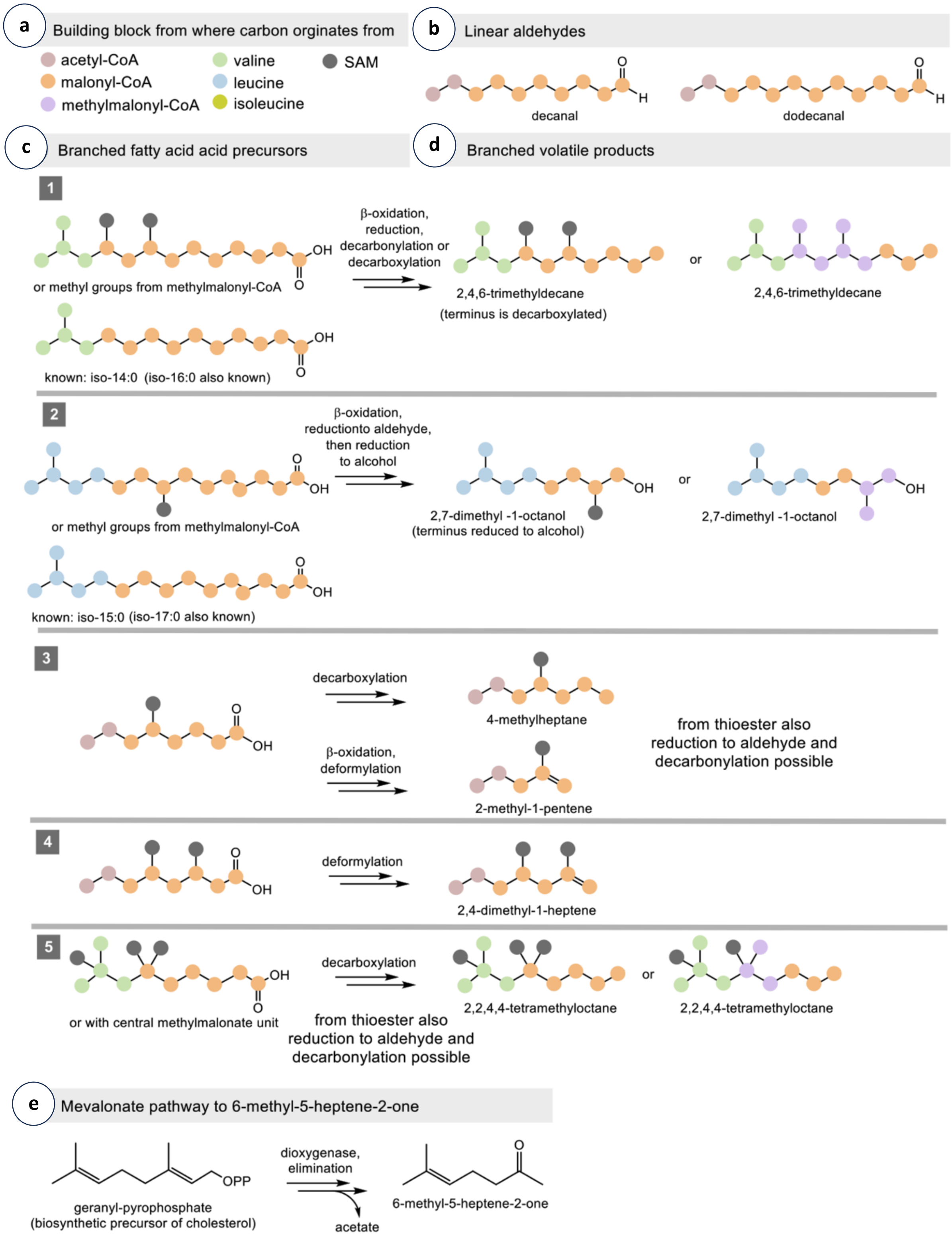

Supplementary Fig. 6 | Proposed biosynthetic origin of individual carbon atoms in fatty acids and geranyl-pyrophosphate derived volatiles reported here (accompanying text see above). (a) Carbon atom colour code of building blocks. (b) Proposed biosynthetic origin of carbon atoms in decanal and dodecanal; (c) Possible precursor fatty acids and biosynthetic origin of carbon atoms;

1: for 2,4,6-trimethyl decane; 2: for 2,7-dimethyl-1-octanol; 3: 4-methylheptane and 2-methyl-1-pentene; 4: 2,4-dimethyl-1-heptene; 5: 2,2,4,4-tetramethyloctane. (d) Proposed biosynthetic origin of carbon atoms in methyl branched VOCs and link to C.: 1: for 2,4,6-trimethyl decane; 2: for 2,7-dimethyl-1-octanol; 3: 4-methylheptane and 2-methyl-1-pentene; 4: 2,4-dimethyl-1-heptene; 5: 2,2,4,4-tetramethyloctane. (e) Geranyl-pyrophosphate, the proposed biosynthetic precursor of 6-methyl-5-hepten-2-one.

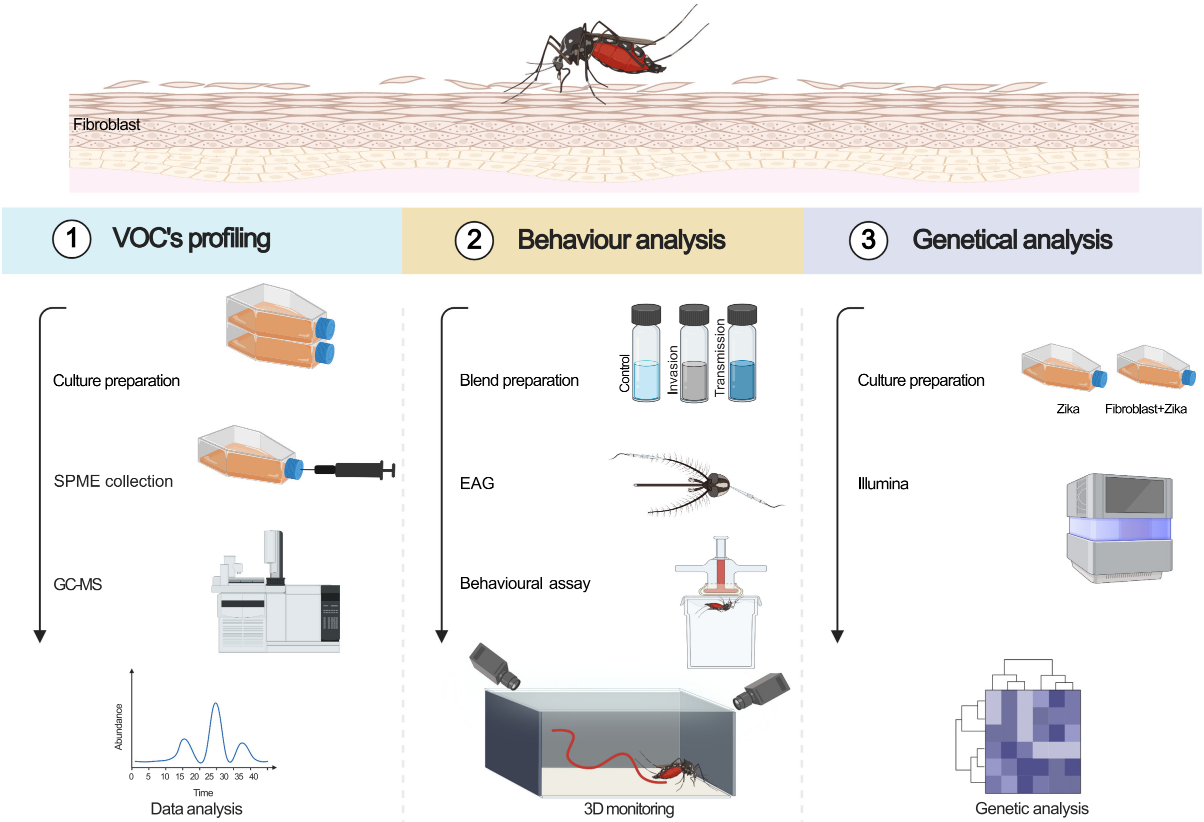

Supplementary Fig. 7 | Schematic model of ZIKV modulates human skin cells to enhance transmission success. (1) ZIKV induces an increased release of volatile organic compound (VOC) blends at two different stages in the fibroblast tissue culture. The VOCs at invasion (10 hpi) and transmission (24 hpi) stages of the virus cycle in human skin cell tissue culture were profiled. This causes (2) an increased attraction of female mosquitoes. The female blood ingestion size and biting rate increase, as well as its fecundity and survival. (3) The behavioural and physiological effects are reflected in the fibroblast transcriptional profile, in which genes/proteins/enzymes involved in ISGF3 complex and lipoprotein metabolic processes as well as immunity are affected. Altogether these alterations increase the probability of virus transmission.

Supplementary Table 1 | Statistical description among volatile profiles in various culture comparisons (A-E)

A	Comparison of Invasion stage vs. cells			
Compound	Estimated value	SD	Pr(>|t|)	Significancy
4-Methyl-heptane(4)	12277211	3367770	0.002201	**
2,4-Dimethyl-1-heptene(10)	63845321	34646932	0.00264	**
Sulcatone(37)	4919287	31571236	0.027336	*
Branched hydrocarbon(53)	5447410	3668800	0.01284	*
Branched hydrocarbon(58)	18 385 930	12145076	1.64e-12	***
Decanal(80)	2131195	2 156 726	0.00609	**
Dodecanal(109)	7647497	4037776	2.499	*
Branched hydrocarbon(54)	1931972	893 841	0.006233	**
Branched hydrocarbon(60)	10897367	11474299	1.17e-06	***
Branched hydrocarbon (64)	9556317	11 186 616	5.52e-05	***
Branched hydrocarbon (65)	9595122	11 202 180	5.10e-05	***
Unidentified(94)	2425195	1 715 443	0.064617	.
Unidentified(101)	1210995	502358	0.065061	.
Nonanal(62)	2498451	6674885	0.0621	.
Branched hydrocarbon(44)	-51404665	-59190709	0.068738	.
2-Nonanone (59)	-11026090	-10455888	1.14e-08	***
Unidentified(61)	-10131205	-10708137	1.01e-06	***
2-Isopropyl-5-methylhex-2-enal(63)	-7579101	-8 231 184	1.57e-06	***
Branched hydrocarbon (66)	-4436614	-10 525 750	4.04e-06	***

B	Comparison of Transmission stage vs. cells			
Compound	Estimated value	SD	Pr(>|t|)	Significancy
2-Methyl-1-pentene(1)	8536460	1997591	1.710	*
Sulcatone(37)	1,58E+07	36471871	2.172	*
Unidentified(49)	239600	3089325	1.824	*
Unidentified(67)	636800	1960978	2.945	**
Branched hydrocarbon(68)	768703	1893104	2.447	*
3-Methyl-undecane(72)	1050501	2097310	2.253	*
Decanal(80)	6363266	2156726	3.633	***
Dodecanal(109)	10458901	5210047	3.106	**

C	Comparison of Invasion stage vs. Transmission stage			
**Compound**	**Estimated value**	**SD**	**Pr(>|t|)**	**Significancy**
2-Methyl-1-pentene(1)	12718942	408530	2.041	*
2,4-Dimethyl-1-heptene(10)	20512140	62444611	-3.404	***
4-Methyl-2-heptanone(23)	4101146	7004252	-3.495	***
Branched hydrocarbon(38)	38686542	-31589837	-2.224	*
Unidentified(49)	449312	1219160	6.246	***
Branched hydrocarbon(50)	1499914	2360709	6.176	***
Branched hydrocarbon(51)	625983	1287158	6.329	***
Unidentified(52)	43117	581148	6.424	***
Branched hydrocarbon(53)	2893452	5657122	4.710	***
Branched hydrocarbon(54)	2131803	3465826	5.811	***
p-Menthan-8-ol(55)	704625	717854	6.828	***
Branched hydrocarbon(58)	9518338	18385930	-7.160	***
Unidentified(67)	1306970	1335787	-1.989	*
Branched hydrocarbon(68)	884438	1580978	-2.396	*
3-Methyl-undecane(72)	1166235	2038699	-3.027	**
Dodecane(78)	6952343	10102882	-1.981	.
Unidentified(47)	-8837459	-8881600	-9.671	***
Benzeneacetaldehyde(48)	-30515	-34028	6.841	***
Unidentified(56)	-16599	-3146	6.828	***
2-Nonanone(59)	-17249079	-17266943	5.073	***
Branched hydrocarbon(60)	-17659886	-17138221	4.765	***
Undecane(61)	-16993406	-16409684	4.730	***
Nonanal(62)	-17606898	-8739306	4.998	***
2-Isopropyl-5-methylhex-2-enal(63)	-22687546	-13819954	4.790	***
Branched hydrocarbon(64)	-24664764	-15797172	4.382	***
Branched hydrocarbon(65)	-16888312	-15835976	4.462	***
Branched hydrocarbon(66)	-24732076	-15864484	4.392	***

D	Comparison of Cells vs. Media			
**Compound**	**Estimated value**	**SD**	**Pr(>|t|)**	**Significancy**
Octane(9)	6630680	14969264	-2.564	*
Styrene(17)	439026	34387436	-5.569	***
Sulcatone(37)	4650014	36471871	-2.254	*
Branched hydrocarbon(38)	2114628	59588598	-2.670	**
2-Ethyl-1-hexanol(46)	128694805	125042655	2.008	*
Branched hydrocarbon(50)	79910	2612996	-2.453	*
Unidentified (98)	391112	1044962	-3.737	***
Dodecane(78)	2573899	9080283	-6.479	***
Unidentified (96)	612707	2580899	-5.287	***
2,6-di-tert-butyl-p-Benzoquinone(115)	19007208	238566	16.483	***
5-Methyl-2-phenyl-2-hexenal(118)	23269228	11673	8.726	***
Ethyl benzene(132)	14749644	308542	5.805	***
2,4-Dimethyl-1-heptene(10)	837872	34646932	5.903	***
Unidentified(49)	253853	3089325	-3.101	**
Branched hydrocarbon(58)	1181252	7357393	-12.367	***
Branched hydrocarbon(60)	11963098	11474299	8.244	***
Undecane(61)	11389002	10708137	8.048	***
Branched hydrocarbon(64)	10262797	11202180	9.707	***
Unidentified(11)	14755954	14520443	1.762	.
Branch hydrocarbon(15)	14969265	14492739	1.710	.
Unidentified(47)	59484415	59526514	1.887	.
Branched hydrocarbon(43)	-59077328	-52916634	1.686	.
Limonene(45)	-59470099	-59493640	1.889	.
1,2,4-trimethyl- Benzene(39)	-59024559	-59111756	1.891	.
Cyclohexanol(16)	-10401398	-11909598	2.141	*
Nonane(18)	-12786221	-14058324	2.090	*
2-Nonanone(59)	-10967564	-10455888	8.221	***
2-Isopropyl-5-methylhex-2-enal(63)	-3142899	-8231184	8.048	***
Branched hydrocarbon(65)	-11707002	-10525750	7.535	***
Nonanal(62)	-7270818	- 6674885	23.028	***
Decanal(80)	-9559062	-2156726	8.456	***
Unidentified (101)	-1386742	-502358	3.019	**

E	Comparison of Virus vs. Media			
**Compound**	**Estimated value**	**SD**	**Pr(>|t|)**	**Significancy**
Unidentified(56)	1417756	-738842	3.210	**
Branched hydrocarbon(57)	1708821	3124860	-2.046	*
2-Isopropyl-5-methylhex-2-enal(63)	7635121	5395747	2.922	**
Dodecanal(109)	18487082	9233328	3.738	***
Unidentified(135)	3344032	474588	2.842	**
4-Methyl-heptane(4)	758338	4144471	1.943	·
Nonadecane(136)	369004	474588	1.936	·
Heneicosane(137)	315446	395430	-1.954	·
2,6-di-tert-butyl-p-Benzoquinone(115)	-8377834	-4199130	-1.700	·
Unidentified(34)	-188315	-1279617	-1.919	·
Unidentified(32)	-2649520	-1344442	-2.282	*
4-Methyl-2-heptanone(23)	-2193317	-3971905	-2.650	**
Unidentified(101)	-703153	-1539298	-2.484	*

Supplementary Table 2 | All statistical descriptions for mosquito fitness studies, A) Fig 2 B) Fig 3 b-c.

A	χ2	P
**Transmission x10**	10.14	0.001
**2-Methyl-1-pentene**	8.90	0.003
**2,4-Dimethyl-1-heptene**	7.20	0.007
**Decanal**	6.02	0.014
**Dodecanal**	15.45	<0.001
**Sulcatone**	7.20	0.007
**Invasion x10**	10.18	0.001
**4- methyl-heptane**	9.72	0.002

B	χ2	P
**Hematin**	50.60	<0.001
	**Z**	**P**
**con/in**	3.15	<0.001
**in/trans**	4.62	<0.001
**con/trans**	7.01	<0.001
	**χ2**	**P**
**Feeding proportion**	106.62	<0.001
	**Z**	**P**
**con/in**	4.25	<0.001
**in/trans**	4.59	<0.001
**con/trans**	6.59	<0.001

Supplementary Table 3 | Enzymes potentially involved in the processing of fatty acids, lipids, isoprenoids and the production of VOCs.

No.	Enzyme
1	3-ketoacyl-coA thiolase
2	3-L-hydroxyacyl-coA dehydrogenase
3	3-oxoacyl-[acyl-carrier-protein] reductase
4	Alcohol dehydrogenases
5	Aldehyde deformylating oxygenases
6	Aldehyde reductase
7	Aldo-keto reductase family 1 member A1
8	Aldo-keto reductase family 1 member B1
9	Carboxylic acid decarboxylases
10	Carboxylic acid reductase
11	Enoyl-coA hydratase
12	Fatty acyl-coA dehydrogenases
13	Inositol oxygenase
14	Oxidative decarboxylases
15	α-Dioxygenase

Supplementary Table 4 | Publications took into account for meta-analysis combining ZIKV infection and mass spectrometry-based shot-gun proteomics on human cells.

No.	FirstAuthor	PMID
1	Wichit *et al.*	30959732
2	Contreras *et al.*	36680182
3	Borges-Vélez *et al.*	36429055
4	Sosa-Acosta *et al.*	34676661
5	Rosa-Fernandes *et al.*	34582966
6	Beys-da-Silva *et al.*	30377986
7	Rosa-Fernandes *et al.*	30949028
8	Shah *et al.*	30550790
9	Scatturro *et al.*	30177828
10	Garcez *et al.*	28112162
11	Legros *et al.*	32817655
12	Jiang *et al.*	29922247

Supplementary Table 5 | Enzymes are affected by ZIKV infection and are potentially involved in the processing of fatty acids, lipids, isoprenoids and the production of VOCs (results of meta-proteome-analysis). The listed ID is related to the full dataset in Extended data file 4 IX

PMID	Cells	Conditions / Read Out	Protein Names	Effect	Gene Names	ID
30177828	Neural Progenitors, Differentiation	Interaction with any ZIKV proteins (AP–MS)	Long–chain enoyl–CoA hydratase; Long chain 3–hydroxyacyl–CoA dehydrogenase	Interaction	HADHA	55
30177828	Neural Progenitors, Differentiation	Interaction with any ZIKV proteins (AP–MS)	3–ketoacyl–CoA thiolase	Interaction	HADHB	75
30177828	Neural Progenitors, Differentiation	Changes upon NS4B overexpression in NPCs and differentiated cells	Acyl–CoA dehydrogenase family member 11	Down	ACAD11	505
30177828	Neural Progenitors, Differentiation	Changes upon NS4B overexpression in NPCs and differentiated cells	UDP–glucuronic acid decarboxylase 1	Up	UXS1	901
30177828	Neural Progenitors, Differentiation	Changes at different time points upon ZIKV infection	Aldo–keto reductase family 1 member C2	Up	AKR1C2	3619
30177828	Neural Progenitors, Differentiation	Changes at different time points upon ZIKV infection	Aldo–keto reductase family 1 member C3	Up	AKR1C3	3827
34582966	Placenta Explants (chorionic villi)	Changes upon ZIKV infection	Aldo–keto reductase family 1 member B1	Up	AKR1B1	4734
30377986	Human Mesenchymal Stem Cells	Changes upon ZIKV infection	Aldo–keto reductase family 1 member C3	Up	AKR1C3	4875
30377986	Human Mesenchymal Stem Cells	Changes upon ZIKV infection	Very long–chain specific acyl–CoA dehydrogenase, mitochondrial	Down	ACADVL	4918
30949028	Neurospheres	Changes upon ZIKV infection (ZIKV–AF vs ZIKV–BR vs MOCK)	Enoyl–CoA hydratase, mitochondrial	Down; Down	ECHS1	5475
30949028	Neurons	Changes upon ZIKV infection (ZIKV–BR vs MOCK, Neurons)	Alcohol dehydrogenase class–3	Up	ADH5	8274
30949028	Neurospheres	Changes upon ZIKV infection (ZIKV–AF vs ZIKV–BR vs MOCK)	3–hydroxyacyl–CoA dehydrogenase type–2	Down; Down	HSD17B10	5412
30949028	Neurospheres	Changes upon ZIKV infection (ZIKV–AF vs ZIKV–BR vs MOCK)	Short/branched chain specific acyl–CoA dehydrogenase, mitochondrial	Down; Up	ACADSB	5413
30949028	Neurospheres	Changes upon ZIKV infection (ZIKV–BR vs MOCK)	3–hydroxyacyl–CoA dehydrogenase type–2	Down	HSD17B10	6300
30949028	Neurons	Changes upon ZIKV infection (ZIKV–BR vs MOCK, Neurons)	Very long–chain specific acyl–CoA dehydrogenase, mitochondrial	Up	ACADVL	7969
30949028	Neurons	Changes upon ZIKV infection (ZIKV–BR vs MOCK, Neurons)	3–hydroxyacyl–CoA dehydrogenase type–2	Up	HSD17B10	8246
30550790	HEK293T	Interaction with ZIKVug NS4A	Retinol dehydrogenase 14 (Alcohol dehydrogenase PAN2)	Interaction	RDH14	9190
30550790	HEK293T	Interaction with ZIKVfp NS4A	UDP–glucuronic acid decarboxylase 1 (UDP–glucuronate decarboxylase 1)	Interaction	UXS1	8881
30550790	HEK293T	Interaction with ZIKVfp NS4A	Acyl–CoA dehydrogenase family member 9, mitochondrial	Interaction	ACAD9	8889
28112162	Neurospheres	Changes upon ZIKV infection	Very long–chain specific acyl–CoA dehydrogenase, mitochondrial	Down	ACADVL	9470
28112162	Neurospheres	Changes upon ZIKV infection	Acyl–CoA dehydrogenase family member 9, mitochondrial	Up	ACAD9	9772
32817655	Muscle Cells	Changes upon ZIKV infection	Acyl–CoA dehydrogenase family member 9, mitochondrial	Up	ACAD9	9935

Supplementary information

Supplementary Figs 1 to 7

Fig. 1: Volatile organic compounds (VOCs) which amounts differ significantly in pairwise comparisons.

Fig. 2: Electroantennographic (EAG) responses of the *Ae. aegypti* female mosquitoes to seven commercially available synthetic VOCs.

Fig. 3: Distinct effects of Zika virus on temporal gene regulation in human skin fibroblasts.

Fig. 4: Proteome analysis of ZIKV infected and primary fibroblasts.

Fig. 5: Fatty acid metabolism that yields alkanes and alkenes by combinations of reductive steps and removal of the carboxylate terminus are summarized and exemplified for decanoate-SCoA.

Fig. 6: Proposed biosynthetic origin of individual carbon atoms in fatty acids and geranyl-pyrophosphate derived volatiles.

Fig. 7: Schematic model of ZIKV modulates human skin cells to enhance transmission success.

Supplementary Tables 1 to 5:

Table 1: All statistical description among volatile profiles in various culture comparisons (A-E).

Table 2: All statistical description for mosquito fitness studies, A) Fig 2 B) Fig 3 B-C

Table 3: Enzymes potentially involved in the processing of fatty acids, lipids, isoprenoids and the production of VOCs

Table 4: Publications took into account for meta-analysis combining ZIKV infection and mass spectrometry-based shot-gun proteomics on human cells.

Table 5: Enzymes are affected by ZIKV infection and are potentially involved in the processing of fatty acids, lipids, isoprenoids and the production of VOCs (results of meta-proteome-analysis). The listed ID is related to the full dataset in Extended Data-file S3 V

Supplementary Data files 1 to 4

file 1: Raw data regarding the VOCs GS-MS analyses

file 2: RNAseq analyses

file 3: Proteome analysis

file 4: VOCs, pathways, and enzymes, meta-proteome
