# Supplementary material for: Zika virus modulates human fibroblasts to enhance transmission success in a controlled lab-setting
Source: Commun Biol. 2025 Jan 30;8:139. doi: 10.1038/s42003-025-07543-9 (PMC11782651; doi:10.1038/s42003-025-07543-9)
Supplement: Supplementary file 6 — Description of Additional Supplementary Files [file 42003_2025_7543_MOESM6_ESM.docx]

Description of Additional Supplementary Files

**File name:** Supplementary data 1

**Description:** Raw data regarding the VOCs GS-MS analyses.

**File name:** Supplementary data 2

**Description:** RNAseq analyses.

**File name:** Supplementary data 3

**Description:** Proteome analysis.

**File name:** Supplementary data 4

**Description:** VOCs, pathways, and enzymes, meta-proteome.
